# New cytogenetic data for some Palaearctic species of scale insects (Homoptera, Coccinea) with karyosystematic notes

**DOI:** 10.3897/CompCytogen.v5i5.2116

**Published:** 2011-12-22

**Authors:** I.A. Gavrilov-Zimin

**Affiliations:** 1 Zoological Institute Russian Academy of Sciences, St. Petersburg, Russia

**Keywords:** scale insects, karyotypes, chromosome numbers, karyosystematics

## Abstract

New cytogenetic data are reported for 17 species from 15 genera of the families Pseudococcidae, Eriococcidae, Kermesidae, and Coccidae. Twelve species and 6 genera (*Peliococcopsis* Borchsenius, 1948, *Heterococcopsis* Borchsenius, 1948, *Heliococcus* Šulc, 1912, *Trabutina* Marchal, 1904, *Lecanopsis* Targioni Tozzetti, 1868, and *Anapulvinaria* Borchsenius, 1952) were studied cytogenetically for the first time. The taxonomic problems in the genera *Trionymus* Berg, 1899, *Acanthopulvinaria* Borchsenius, 1952 and *Rhizopulvinaria* Borchsenius, 1952 are discussed based on karyotype characters. Two chromosomal forms (cryptic species) of *Acanthopulvinaria orientalis*(Nasonov, 1908), 2n=18 and 2n=16 were discovered.

## Introduction

In June 2009 the author and Dr. Mehmet Bora Kaydan made several joint collecting trips in Eastern Anatolia (Turkey). Part of the material collected during these trips, plus some other material collected by M.B. Kaydan without me in 2009, proved to be suitable for cytogenetic studies. Turkey in general and especially Eastern Anatolia have an exceptionally rich scale insect fauna (Kaydan and Kozár 2010, 2011, Scalenet: http://www.sel.barc.usda.gov/scalenet/scalenet.htm, accessed on 14 September, 2011) not only in terms of species and genera diversity (more then 300 species, 117 genera have been recorded for Turkey), but also in terms of populations density. The latter fact is especially important for species, living on roots of wild plants. Most of these species, usually rarely collected in other Palaearctic regions as single adult females from one or two collecting points can be found in Eastern Anatolia comparatively easily as numerous females (and often with males and larvae of both sexes) in numerous localities. The high density of populations, in turn, is especially important for cytogenetic studies which often demand a high number of prepared insects. Hereby material collected in Eastern Anatolia provides a good possibility to clarify cytogenetic characteristics not only for newly studied species but also for some species that were insufficiently studied earlier.

Until now, Palaearctic scale insects were studied cytogenetically rather fragmentarily and significantly more poorly than tropical and subtropical species (Gavrilov 2007a). However, the available data (mainly from the author’s previous papers) and comparison of these data with the new information reported here allow generation of some karyotaxonomic conclusions (see below).

The unique genetic systems of scale insects (*XX-X0, n-2n (Haplo-diploidy), Hermaphroditism, 2n-2n, Lecanoid, Comstockioid, Diaspidoid, obligate Thelytoky*) have been reviewed many times in special papers (Schrader 1923a, 1929, Brown 1958-1969, Hughes-Schrader 1948, Nur 1962-1990, Haig 1993, Normark 2003, Gavrilov 2007a, Gavrilov and Kuznetsova 2007) and so will not be discussed here, except only for the following detail. Nur (1980), based on his own studies and literature data, noted that the Comstockioid genetic system differs from the Lecanoid system “…in the destruction or loss of from one to all the H chromosomes just prior to prophase I of spermatogenesis”. This approach assumes that it is impossible to distinguish the Lecanoid and the Comstockioid systems without analysis of spermatogenesis. In practice the collecting of third-instar larvae of males (stage of spermatogenetic divisions) or even males themselves is a very rare event for most scale insect species. Even if these larvae are collected it is often rather difficult to prepare good slides of male meiotic chromosomes because of difficulties with the methods of squashing testis tissue. On the other hand, based on my long term work with scale insect chromosomes, it seems that the Comstockioid genetic system is visually different in easily studied male embryonic cells: heterochomatic elements usually do not form compact singular heterochomatic body in interphase nuclei (Fig. 21) (in contrast to the Lecanoid system) and at least some cells have fewer heterochomatic chromosomes than the haploid number (as, for example, on Fig. 19). According to this indirect evidence it may be possible to note Lecanoid or Comstockioid heterochromatinization for newly studied species and genera of the higher taxa for which Lecanoid or Comstockioid systems were previously detected by studies of spermatogenesis. In the present paper this admission was made for species of the families Pseudococcidae, Eriococcidae and Kermesidae.

Some scale insects (in particular, some of those listed below) are characterized by a unique individual development that is similar to a double fertilization in angiosperms. In this case each embryo develops from two different cells. One of those is a normal diploid zygote that gives rise to the majority of tissues. The other cell is a polyploid secondary zygote that results from the fusion of a cleavage nucleus with the first or second polar bodies. The secondary zygote gives rise to the polyploid bacteriome (or mycetome). Each cell of the bacteriome (or mycetome) thus includes one haploid set of paternal chromosomes and several maternal sets (Schrader 1923b, Hughes-Schrader 1948, Brown 1965, Normark 2001, Gavrilov 2007a). This phenomenon has been studied mainly in Diaspididae and Pseudococcidae, which can display 5-ploid, 7-ploid or even 14-ploid bacteriomes (Brown 1965, Normark 2004). It is not known whether other coccid families also have “dizigotic soma” or other mechanisms of bacteriome-formation similar to some soft scales (Tremblay 1961) or to the genus *Puto* (Pseudococcidae s.l.) (Brown and Cleveland 1968).

## Material and methods

All material for this study was collected in 2009 in Eastern Anatolia (Turkey). The detailed collecting data are listed below, separately for each species in order to avoid the double citations of taxonomic names and for more comfortable using of the paper.

The chromosomal plates were made as previously described (Gavrilov and Trapeznikova 2007, 2008).

All material is deposited at the Zoological Institute, Russian Academy of Sciences, St. Petersburg.

## Results and discussion

### Family Pseudococcidae

***Puto superbus* (Leonardi, 1907)**

Figs 1–3

*Material*. K 607, Igdir-Digor road, 40°10'451"N, 43°40'389"E, on steams of grass, 04.06.2009, M.B. Kaydan & I. Gavrilov.

Embryos from female body. 2n=16 +XX (♀), 2n= 16 +X0 (♂).

Hitherto, only American species of the genus *Puto* Signoret, 1876 were studied cytogenetically (Hughes-Schrader 1944, Brown and Clevelend 1966). *Puto superbus* is the first studied species of the genus from the Palaearctic fauna; it also has an ancient XX/X0 genetic system (as 5 other studied species of the genus), but demonstrates a different chromosome number (2n=18/17) in contrast to 2n=14/13, 16/15, 20/19 in American species.

**Figures 1–3.**
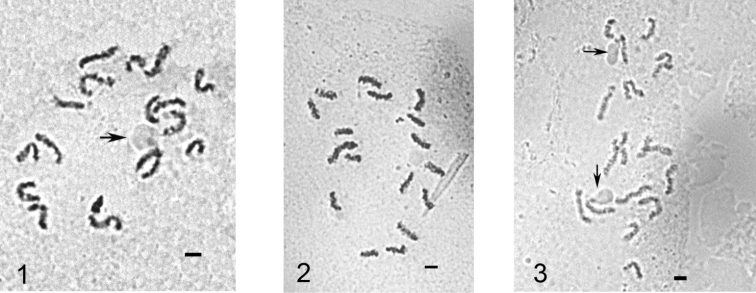
Mitotic chromosomes of *Puto superbus*. **1** cell of female embryo, 2n=18 **2, 3** cells of male embryo, 2n=17. The chromosomes with NORs are arrowed. Bar = 10 µm.

Figures 1 & 3 illustrate nucleoli localized at the ends of the middle-sized chromosomes. The localizations of NORs in scale insects were discussed earlier by Gavrilov 2005, Gavrilov and Trapeznikova 2007 based their own and literature data. *Puto superbus* shows the new pattern of this localization in contrast to the position of NORs on longest or shortest chromosomes in other studied coccid species.

***Phenacoccus* Cockerell, 1893**

Hitherto, 16 species of the large and widely distributed genus *Phenacoccus* have been studied by different authors (see the review of Gavrilov 2007a and Gavrilov and Trapeznikova 2007, 2010). Most of studied species demonstrate the modal chromosomal number 2n=10. Here I am adding the data on 3 species, which have not been studied before.

Sharing the same chromosomal number *Phenacoccus* spp. demonstrate, however, significant variation of chromosomal lengths in their karyotypes. This variation in combination with the data on differential staining of *Phenacoccus* spp. chromosomes will probably provide the basis for further karyotaxonomic studies of the genus.

***Phenacoccus specificus* Matesova, 1960**

Fig. 4

*Material*. K 603 (4472), Kars - Kagizman road, 40°16'351"N, 42°52'275"E, 1761 m alt., on roots of *Thymus* sp., 04.06.2009, M.B. Kaydan & I. Gavrilov.

Embryos from female body. 2n=10.

**Figures 4–7.**
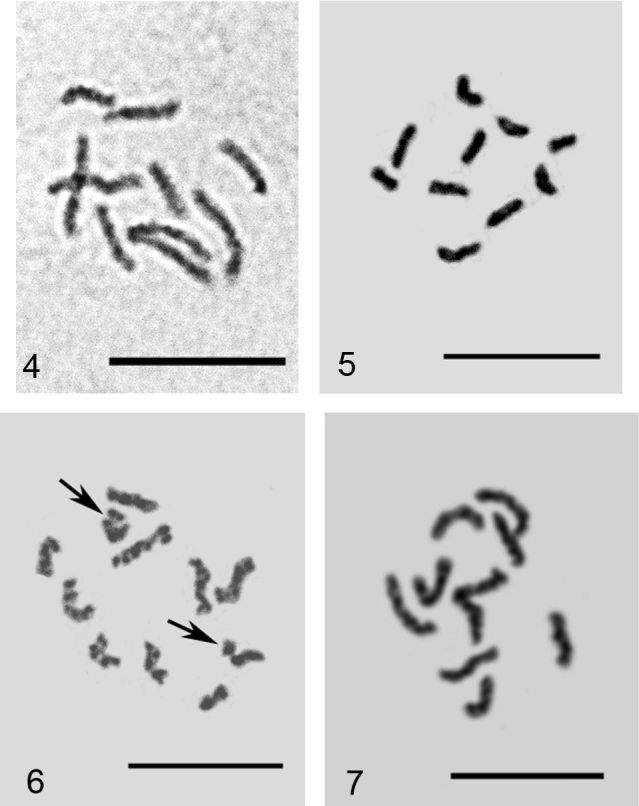
Embryonic cells of *Phenacoccus* spp. **4** *Phenacoccus specificus*, 2n=10 **5, 6** *Phenacoccus phenacoccoides*: **5** 2n=10, **6** 2n=10+Bs, additional chromosomal elements arrowed **7** *Phenacoccus tergrigorianae*, 2n=10. Bar = 10 µm.

***Phenacoccus phenacoccoides* (Kiritshenko, 1932)**

Figs 5–6

*Material*. K 612 (4483), Kars-Kagizman road, 40°12'011"N, 43°02'827"E, 1273 m alt., under the leaf sheaths of grass, 04.06.2009, M.B. Kaydan & I. Gavrilov.

Embryos from female body. 2n=10, 2n=10 + Bs, Lecanoid heterochromatinization.

The studied population of *Phenacoccus phenacoccoides* demonstrates variation from 0 to 2 additional chromosomal elements (B-chromosomes) between embryonic cells like as seen in a population from the Voronezh region (central part of European Russia) studied earlier by me (Gavrilov 2004).

***Phenacoccus tergrigorianae* Borchsenius, 1956**

Fig. 7

*Material*. K 693, Van-Hakkari road, 37°32'340"N, 43°43'173"E, on roots of undetermined Asteraceae, 02.09.2009, M.B. Kaydan. K 689, the same data, but on *Sorghum halepense*.

2n=10, Lecanoid heterochromatinization.

***Peliococcopsis priesneri* (Laing, 1936)**

Figs 8–9

*Material*. K 601, Agri-Dogubeyazid-Ishakpasa, 39°31'905"N, 44°07'100"E, 2059 m alt., under the leaf sheaths of *Cynodon dactylon*, 03.06.2009, M.B. Kaydan & I. Gavrilov.

Embryos from female body. 2n=10, Lecanoid heterochromatinization.

It is the first species of the genus *Peliococcopsis* Borchsenius, 1948 studied cytogenetically.

**Figures 8–9.**
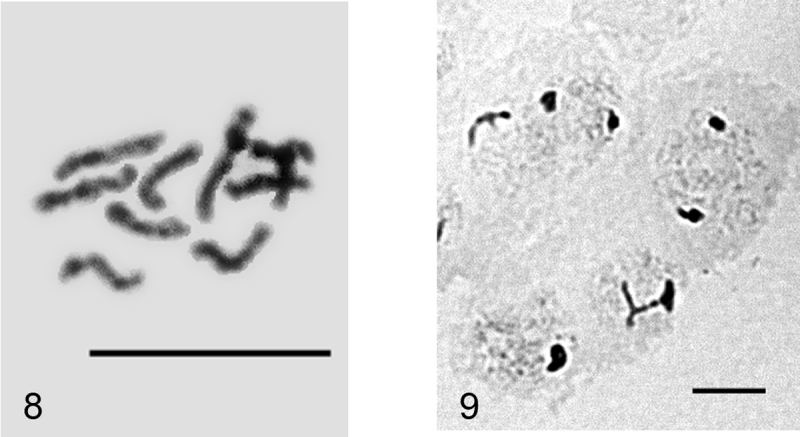
*Peliococcopsis priesneri*. **8** mitotic chromosomes, 2n=10 **9** male embryonic cells at interphase stage with one haploid set heterochomatinized. Bar = 10 µm.

***Heterococcopsis opertus* Borchsenius, 1949**

Fig. 10

*Material*. 4530, Eastern Anatolia without concrete location, 2009, M.B. Kaydan.

Embryos from female body. 2n=10.

It is the first species of the genus *Heterococcopsis* Borchsenius, 1948 studied cytogenetically.

**Figure 10.**
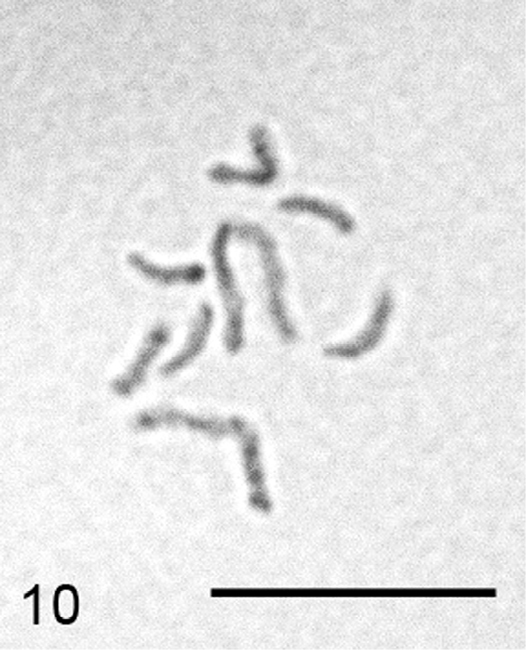
*Heterococcopsis opertus*, embryonic cell, 2n=10. Bar = 10 µm.

***Heliococcus sulci* Goux, 1934**

Fig. 11

*Material*. K677, Hatay-Erzin, 08.09.2009, M.B. Kaydan.

**Figure 11.**
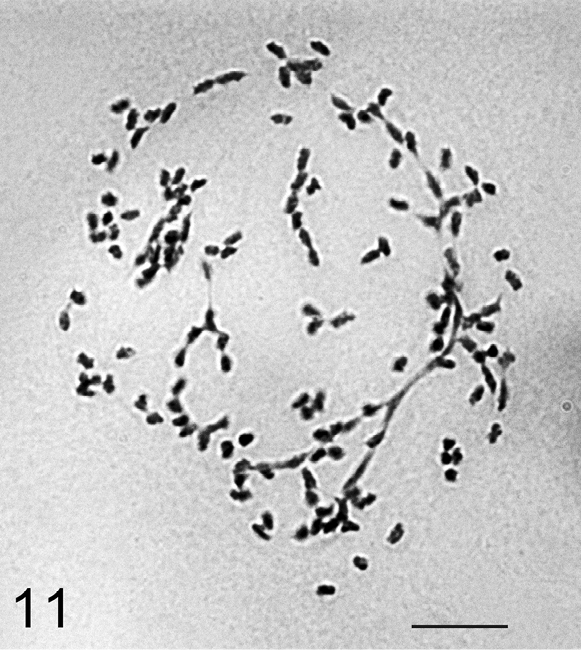
*Heliococcus sulci*, the cell of mycetome, about 140 chromosomes with numerous agglutinations. Bar = 10 µm

Only one female was available for cytogenetic studies and the specimen did not provide cells with chromosome plates suitable for karyotype study. However, some polyploid cells of the mycetome with about 140 chromosomes and numerous agglutinations were observed. In view of the absence till now of any cytogenetic data on the large and very important for phylogenetic reconstructions genus *Heliococcus* Šulc, 1912 I am presenting here the first photograph of *Heliococcus* chromosomes (Fig. 11). It appears that there is no significant size difference between chromosomes.

***Trabutina crassispinosa* Borchsenius, 1941**

Fig. 12

*Material*. K 605, Igdir-Digor road, Kars border, 40°07'278"N, 43°37'708"E, on branch of *Tamarix* sp., 04.06.2009, M.B. Kaydan & I. Gavrilov.

Embryos from the ovisacs. 2n=16.

It is the first species of the genus *Trabutina* Marchal, 1904 studied cytogenetically.

**Figure 12.**
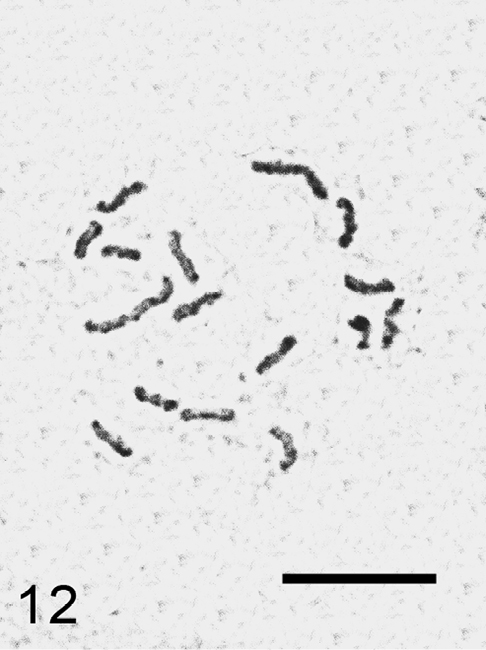
*Trabutina crassispinosa*, cell of embryo, 2n=16. Bar = 10 µm.

***Planococcus vovae* (Nasonov, 1908)**

Figs 13–14

*Material*. K 686, Şanliurfa, on *Cupressus* sp., 09.09.2009, M.B. Kaydan.

2n=10, Lecanoid heterochromatinization. The studied population deviates morphologically from the usual *Planococcus vovae* having 2 circuli in contrast to 1 (or, exceptionally, none) in huge material from different regions of the Palaearctic (Danzig and Gavrilov 2010). However, the karyotype characters seem to be the same as in a previously studied population from the Mediterranean coast of Turkey (Adana) (Gavrilov 2007 and unpublished) that included females with only 1 circulus.

**Figures 13–14.**
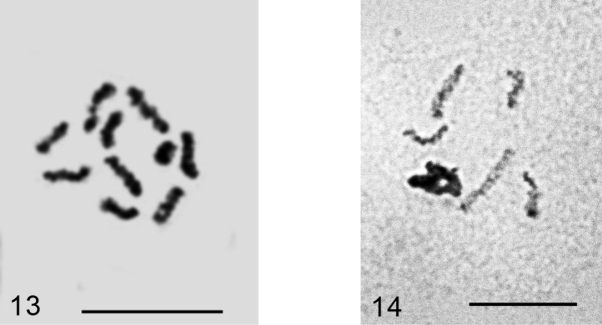
*Planococcus vovae* **13** mitotic chromosomes, 2n=10 **14** male embryonic cell with one haploid set heterochomatinized. Bar = 10 µm.

***Dysmicoccus multivorus* (Kiritshenko, 1936)**

Figs 15–16

*Material*. K 685, Van-Akdamar, on undetermined Apiaceae, 09.06.2009, M.B. Kaydan & I. Gavrilov.

2n=10+1-2 B. All previously studied populations of this species (Gavrilov and Trapeznikova 2007) from the central part of European Russia and Crimea (Ukraine) showed a stable chromosomal number 2n=10. Turkish material shows 1 or 2 additional (B) chromosomes. Mycetocytes with 35 (7x) chromosomes.

**Figures 15–16.**
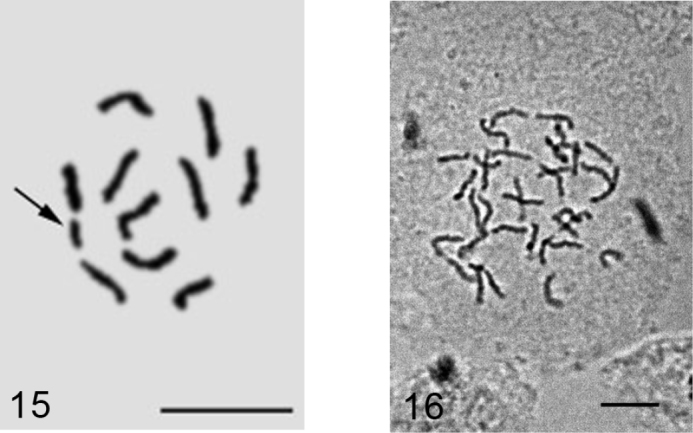
*Dysmicoccus multivorus*. **15** embryonic cell with 2n=10 + B, additional chromosomal element arrowed **16** cell of mycetome, 7x=35. Bar = 10 µm

***Trionymus artemisiarum* (Borchsenius, 1949)**

Fig. 17

*Material*. K 680 (4536), Ağri-Patnos-Adilcevaz road-Aktepe, on *Achillea* sp., 10.06.2009, M.B. Kaydan.

Embryos from female body. However, oviposition takes place during earlier stages of embryonic development, before gastrulation.

2n=10, Lecanoid heterochromatinization.

The species demonstrates another new example of karyotaxonomic rule discovered by the author (Gavrilov 2005, 2007a, Gavrilov and Trapeznikova 2007, 2010) in the genus *Trionymus* Berg, 1899. All species that significantly deviate from morpho-ecological diagnosis of the genus, have chromosome numbers different from the type species, *Trionymus perrisii* (Signoret, 1875); the last one as well as morphologically similar with it *Trionymus aberrans* Goux, 1938 and *Trionymus haancheni* (McKenzie, 1960) have 2n=16.Morphologically deviating *Trionymus multivorus* (Kiritshenko, 1936) and *Trionymus radicum* (Newstead, 1895), both with 2n=10, are considered by me now in the genera *Dysmicoccus* Ferris, 1950 and *Balanococcus* Williams, 1962 correspondingly (Gavrilov 2005, 2007a, Gavrilov and Trapeznikova 2007, 2010). *Trionymus artemisiarum* studied here for the first time also have deviating chromosomes number (2n=10) and deviating classic taxonomic characters. In contrast to other *Trionymus* spp., *Trionymus artemisiarum* has a broadly oval body (not elongate body with parallel margins) and lives on roots of dicotyledonous herbs (not under the leaf sheaths of Poaceae).

**Figure 17.**
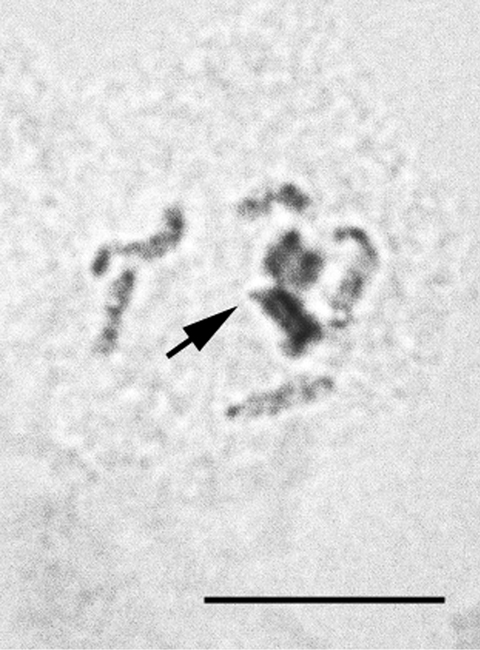
*Trionymus artemisiarum*, 2n=10, cell of male embryo, one haploid set (arrowed) is heterochomatinized. Bar = 10 µm.

### Family Eriococcidae

***Acanthococcus lactucae* Borchsenius, 1949**

Figs 18–19

*Material*. K 675 (4555), Hakkari-Çukurca road, on *Cichorium* sp., 01.09.2009, M.B. Kaydan.

Embryos from female body.

2n=16, heterochromatinization, presumably Comstockioid. The same characteristics have been previously detected in two other species of this genus (Gavrilov 2004, 2007a).

**Figures 18–19.**
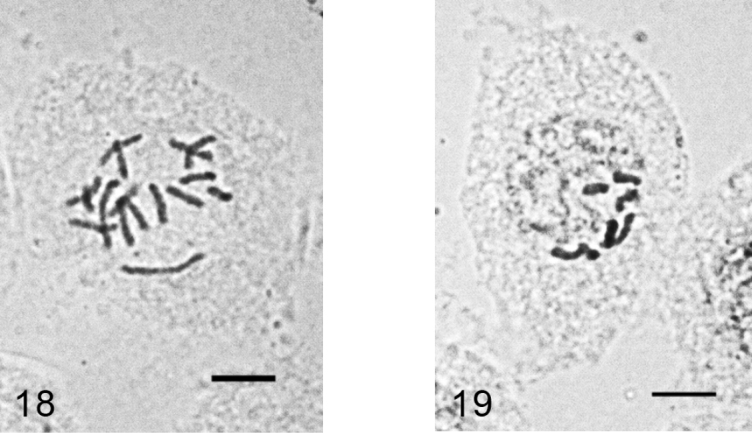
*Acanthococcus lactucae*. **18** mitotic chromosomes in female embryo, 2n=10. **19** - male embryonic cell in interphase with one haploid set heterochomatinized. Bar = 10 µm.

### Family Kermesidae

***Kermes roboris* (Fourcroy, 1785)**

Figs 20–21

*Material*. K 636, Tatvan-Güroymak road, 38°33'187"N, 42°05'851"E, 1570 m alt., on twigs of *Quercus* sp., 10.06. 2009, I. Gavrilov.

Embryos from female body. 2n=26, heterochromatinization, presumably of the Comstockioid type. It is the second studied species of the genus *Kermes* Linnaeus, 1758 and of the whole family Kermesidae. The first studied species, *Kermes quercus* (Linnaeus, 1758), also has 2n=26 and presumable Comstockioid heterochromatinization (Gavrilov 2004, 2007a)

**Figures 20–21.**
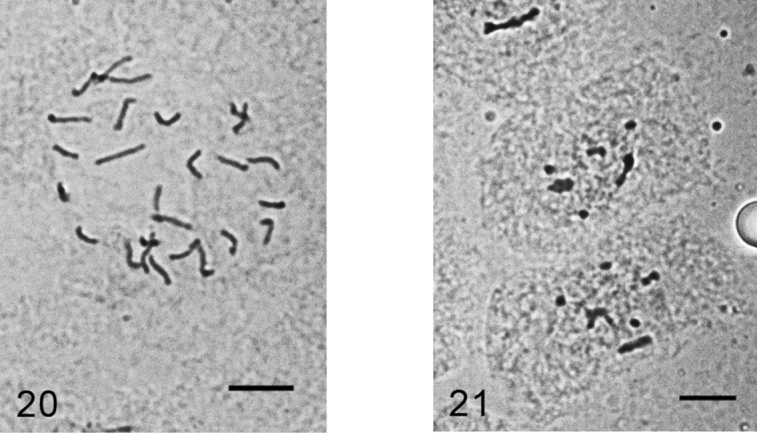
*Kermes roboris*. **20** mitotic chromosomes in female embryo, 2n=26 **21** male embryonic cell in interphase with one haploid set heterochomatinized. Bar = 10 µm.

### Family Coccidae

***Lecanopsis turcica* (Bodenheimer, 1951)**

Figs 22

*Material*. K 592, Dogubeyazit – Igdir road, 39°46'51"N, 44°08'584"E, 1552 m alt., on roots of undetermined Poaceae, 03.06.09, I. Gavrilov.

Embryos from female body. 2n=18, heterochomatinization of an unidentified type. It is the first species of comparatively large Palaearctic genus *Lecanopsis* Targioni Tozzetti, 1868 studied cytogenetically.

**Figure 22.**
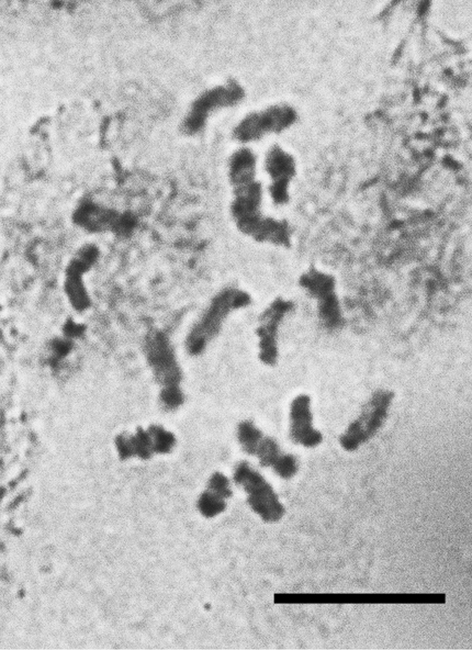
*Lecanopsis turcica*, cell of female embryo, 2n=18. Bar = 10 µm.

***Acanthopulvinaria orientalis* (Nasonov, 1908)**

Figs 23–24

*Material*. K 631 (4502), Van Gurpinar road, on steams of *Artemisia* sp., M.B. Kaydan & I. Gavrilov.

Embryos from ovisacs. 2n=16, heterochromatinization of an unidentified type.

**Figures 23–24.**
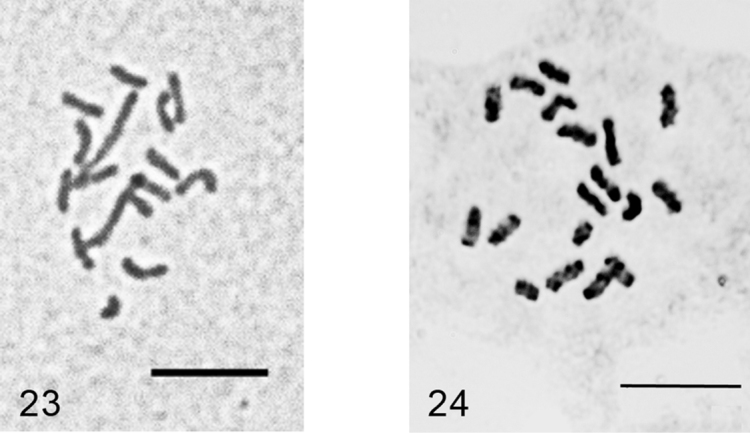
*Acanthopulvinaria orientalis*, cells of female embryo **23** Turkey, 2n=16 **24** Astrakhan (Russia), 2n=18, after Gavrilov 2007a. Bar = 10 µm.

These new data revealed that *Acanthopulvinaria orientalis*, earlier studied from Astrakhan only (Russian coast of the Caspian Sea) (Gavrilov 2007b, Gavrilov and Trapeznikova 2008), hides a minimum 2 chromosomal forms (cryptic species) with 2n=16 and 2n=18 (Figs 23-24). Moreover, 16-chromosome form (present study) demonstrates a pair of extra-large chromosomes that probably originated (in phylogenetic meaning) from a fusion between two chromosome pairs in 18-chromosomal karyotype. It seems that the new chromosomal number does not concern to *Acanthopulvinaria discoidalis* (Hall, 1923), recently placed under synonymy of *Acanthopulvinaria orientalis* (Gavrilov 2007b). *Acanthopulvinaria discoidalis* hasnever been noted anywhere outside of Egypt and has not clear morphological differences from *Acanthopulvinaria orientalis*. The two populations studied by me cytogenetically (Russian and Turkish) also have not structural morphological differences lying outside the usual variability of *Acanthopulvinaria orientalis*. However, Astrakhan females (2n=18) are smaller (about 3 mm long) than the Turkish specimens (2n=16 and about 4 mm long) and both are significantly larger than noted in the original description of Hall (1923) – 1.25–1.5 mm long. It is interesting that in a similar situation with two cryptic species, *Pulvinaria ribesiae* Signoret, 1873 (2n=18) and *Pulvinaria vitis* Linnaeus, 1758 (2n=16), the first one, having higher chromosomal number, is also smaller-sized (Drozdovsky 1966, Gavrilov and Trapeznikova 2008).

Since 2n=16 and 2n=18 chromosomal sets obviously cannot produce fertile hybrid progeny due to meiotic abnormalities they should be treated as two separate species. However, for a final taxonomic decision it is necessary to study more populations from different parts of *Acanthopulvinaria orientalis* geographic area.

***Rhizopulvinaria artemisiae* (Signoret, 1873)**

Figs 25–27

*Material*. K 595 (4462), Çaldiran-Dogubeyazit road, 39°11'71"N, 44°00'784"E, on *Acantholimon* sp., 03.06.09, M.B. Kaydan & I. Gavrilov. K 598 (4467), Agri-Dogubeyazit-Ishakpaşa Palace, 39°31'905"N, 44°07'100"E, 2059 m alt., on *Artemisia* sp., 03.06.09, M.B. Kaydan & I. Gavrilov. K 610 (4481), Kars-Kagizman road, 40°12'011"N, 43°02'827"E, 1273 m alt., on *Artemisia* sp., 04.06.09, M.B. Kaydan & I. Gavrilov.

Embryos from female body. 2n=28, male embryos with heterochromatinization are absent.

**Figures 25–27.**
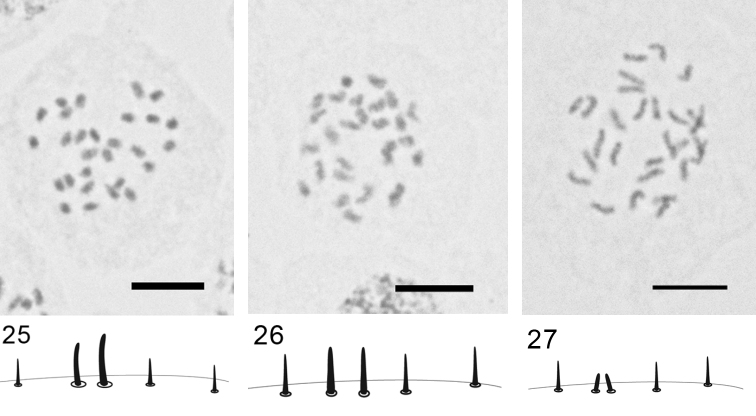
*Rhizopulvinaria artemisiae,*2n=28, embryonic cells and marginal setae **25** population K 595 **26** population K 598 **27** population K 610. Bar = 10 µm

Three Turkish populations studied here show the same karyotype with 28 approximately equal in size chromosomes as in a previously studied population from Astrakhan (Gavrilov 2007a, 2009, Gavrilov and Trapeznikova 2008). These new data confirm the author's conception of polytypic variable species *Rhizopulvinaria arthemisiae* sensu lato (Gavrilov 2009) and the synonymization of numerous nominal species (=forms), described by different authors without any clear differential characters (see the references in the revision of Gavrilov 2009). The studied Turkish populations fortunately show most usual and representative examples of morphological variation of marginal and stigmatic conical setae in *Rhizopulvinaria arthemisiae* s. l. (Figs 25-27) and none the less the karyotype stability, that seems especially important as an additional taxonomic character in view of significant variability of chromosomal number in the Pulvinariini in general.

***Anapulvinaria pistaciae* (Bodenheimer, 1926)**

Figs 28–29

*Material*. K 647, Bitlis, ruins of ancient fortress, on twig of *Pistacia* sp., 10.06.2009, M.B. Kaydan & I. Gavrilov.

Eggs from female body. The laid eggs are of two colors: white and brown.

2n=16?, heterochromatinization.

The monotypic genus *Anapulvinaria* Borchsenius, 1952 was studied here cytogenetically for the first time. Unfortunately, the only female with ovisac was collected and analyzed; the embryos (more than 100 were squashed) demonstrated numerous tripolar mitotic divisions. According to this abnormality and also due to a small number of chromosomal plates suitable for karyotype analysis (2 cells of female embryo and 3 cells of male embryo in total) I am giving the chromosomal number with small doubt. Some embryos contained polyploid cells with about 50 chromosomes.

**Figures 28–29.**
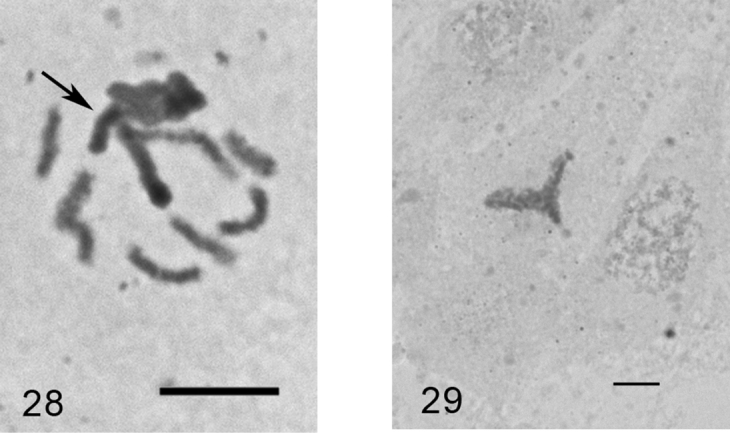
*Anapulvinaria pistaciae,* embryonic cells **28** cell of male embryo with one haploid set heterochromatinized (arrowed) and 8 euchomatic chromosomes **29** tripolar mitosis. Bar = 10 µm.
